# Recommendations for Intraoperative Adverse Events Data Collection in Clinical Studies and Study Protocols. An ICARUS Global Surgical Collaboration Study

**DOI:** 10.29337/ijsp.183

**Published:** 2023-02-09

**Authors:** Giovanni E. Cacciamani, Michael Eppler, Aref S. Sayegh, Tamir Sholklapper, Muneeb Mohideen, Gus Miranda, Mitch Goldenberg, Rene J. Sotelo, Mihir M. Desai, Inderbir S. Gill

**Affiliations:** 1 USC Institute of Urology and Catherine and Joseph Aresty Department of Urology, Keck School of Medicine, University of Southern California, Los Angeles, CA, US

**Keywords:** intraoperative complications, intraoperative adverse event, protocol development, variables, patient data, core set variables, database

## Abstract

**Introduction:**

Intraoperative adverse events (iAEs) occur and have the potential to impact the postoperative course. However, iAEs are underreported and are not routinely collected in the contemporary surgical literature. There is no widely utilized system for the collection of essential aspects of iAEs, and there is no established database for the standardization and dissemination of this data that likely have implications for outcomes and patient safety. The Intraoperative Complication Assessment and Reporting with Universal Standards (ICARUS) Global Surgical Collaboration initiated a global effort to address these shortcomings, and the establishment of an adverse event data collection system is an essential step. In this study, we present the core-set variables for collecting iAEs that were based on the globally validated ICARUS criteria for surgical/interventional and anesthesiologic intraoperative adverse event collection and reporting.

**Material and Methods:**

This article includes three tools to capture the essential aspects of iAEs. The core-set variables were developed from the globally validated ICARUS criteria for reporting iAEs (item 1). Next, the summary table was developed to guide researchers in summarizing the accumulated iAE data in item 1 (item 2). Finally, this article includes examples of the method and results sections to include in a manuscript reporting iAE data (item 3). Then, 5 scenarios demonstrating best practices for completing items 1–3 were presented both in prose and in a video produced by the ICARUS collaboration.

**Dissemination:**

This article provides the surgical community with the tools for collecting essential iAE data. The ICARUS collaboration has already published the 13 criteria for reporting surgical adverse events, but this article is unique and essential as it actually provides the tools for iAE collection. The study team plans to collect feedback for future directions of adverse event collection and reporting.

**Highlights:**

- This article represents a novel, fully-encompassing system for the data collection of intraoperative adverse events.
- The presented core-set variables for reporting intraoperative adverse events are not based solely on our opinion, but rather are synthesized from the globally validated ICARUS criteria for reporting intraoperative adverse events.
- Together, the included text, figures, and ICARUS collaboration-produced video should equip any surgeon, anesthesiologist, or nurse with the tools to properly collect intraoperative adverse event data.
- Future directions include translation of this article to allow for the widest possible adoption of this important collection system.

## Introduction

Intraoperative adverse events (iAEs) are broadly categorized as unintended incidents during a surgical procedure. Though iAEs have been associated with increased morbidity, mortality, and postoperative complication rates [1,2], they have been underreported due to heterogeneous criteria and a lack of widespread acceptance [3,4]. In contrast, postoperative complication reporting has largely increased over the last few decades as a result of standardization and uniformity [5,6,7,8,9], thereby contributing to improved patient safety due to enhanced data quality and accuracy [10,11,12,13,14,15].

Given the link between intraoperative and postoperative adverse events [1,2], the incentive to better understand iAEs is critical, as they offer an innovative lens through which patient care can be advanced. Even though prior attempts have been made, the reporting of iAEs still lacks standardization, resulting in a hurdle for optimal usage [16,17,18,19].

To overcome this barrier, the Intraoperative Complication Assessment and Reporting with Universal Standards (ICARUS) Global Surgical Collaboration is constructing the foundation and resources needed to streamline the adoption and implementation of a standardized definition to assess, collect, grade, and report all iAEs [20,21,22]. This model creates a simple yet organized approach for gathering patient safety data, which not only fosters accurate reporting [5,16], but also creates interstudy reliability and consistency.

Here, we provide a framework for a standardized, transparent, comprehensive, and accurate iAE data collection system that is broadly applicable in surgery and anesthesiology.

## Elaboration and Explanation

This core-set variable proposal represents one of the steps in the development of the ICARUS reporting criteria as previously described in a published protocol [23]. Thirteen iAE collection criteria developed through a modified Delphi consensus panel were previously published [23,24], and those results inform the present study. The ICARUS criteria list is registered in EQUATOR Network Database (https://www.equator-network.org/wp-content/uploads/2022/09/ICARUS-checklist.pdf). This study was reviewed by the intuitional IRB (UP-21-00473), was registered to ClinicalTrials.gov (NCT04994392), and has been performed as part of the assessment for the development of reporting guidelines.

The 13 collection criteria earned high agreement among healthcare providers representing most specialties, anesthesiologists, and interventionalists for clinical usefulness and quality assessment [21]. In a subsequent publication, a ≥80% agreement was reported on their clarity, exhaustiveness, clinical utility, quality assessment, improvement utility, and research utility [25].

**Figure 1:**
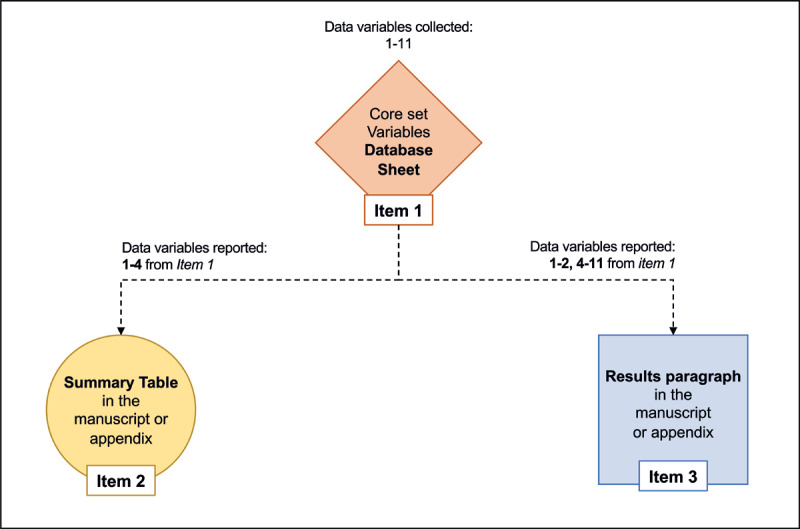
Intraoperative adverse event core set variables development and explanation.

In the present study, the proposed tools to ensure the complete collection and reporting of iAEs that can occur during the surgical/interventional procedure are described (Figure 1). The tools are the core-set variable data table (**item 1**), summary table (**item 2**), and results in summary (**item 3**). Specifically, the reader can download the spreadsheet file with the core-set variables that can be used as the database for proper collection of the intraoperative adverse events (supplemental materials). These tools are available in the supplementary materials. A description of the core-set variables, as well as the rationale for collecting, grading, and reporting iAEs, are described below under item 1.

**Figure 2:**
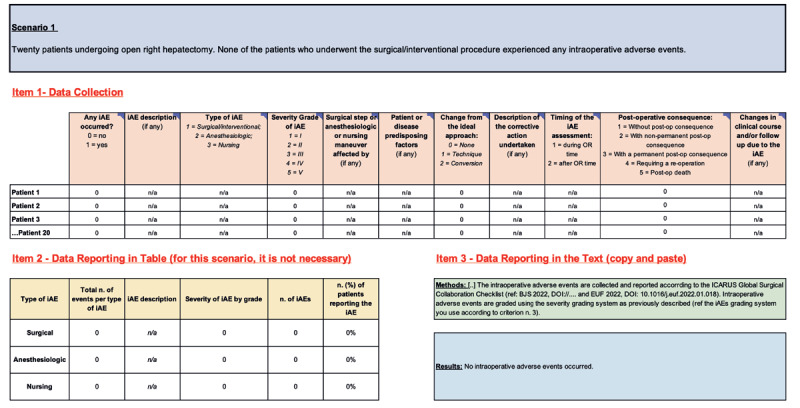
Scenario n.1: No Intraoperative Adverse Events Occurred. iAEs: Intraoperative Adverse Events; Post-op: Post-operative; n/a: Not applicable.

Additionally, below are 5 iAE data collection scenarios (Figure 2,, 3, 4, 5, 6) to promote best practices when utilizing these newly developed tools. For improving adherence to the ICARUS recommendation for iAEs collection, we have produced a video course explaining how to complete **items 1–3** for the 5 iAE data collection scenarios (https://youtu.be/pFaU7AJVLIs).

**Figure 3:**
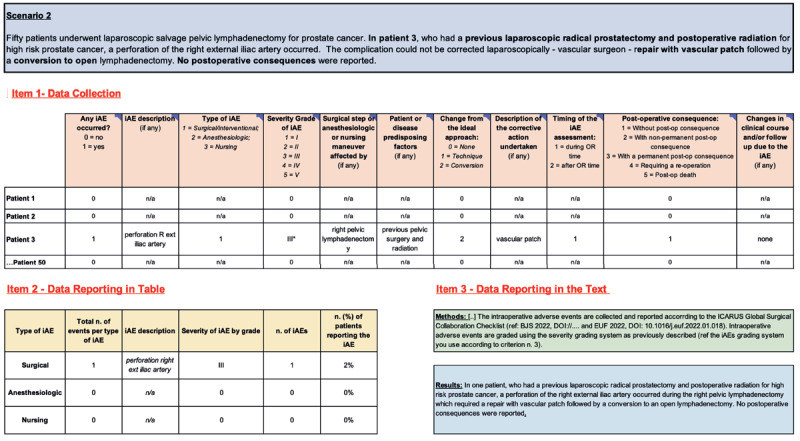
Scenario n.2: One Intraoperative Adverse Event in One Patient. iAEs: Intraoperative Adverse Events; Post-op: Post-operative; Not applicable.

**Figure 4:**
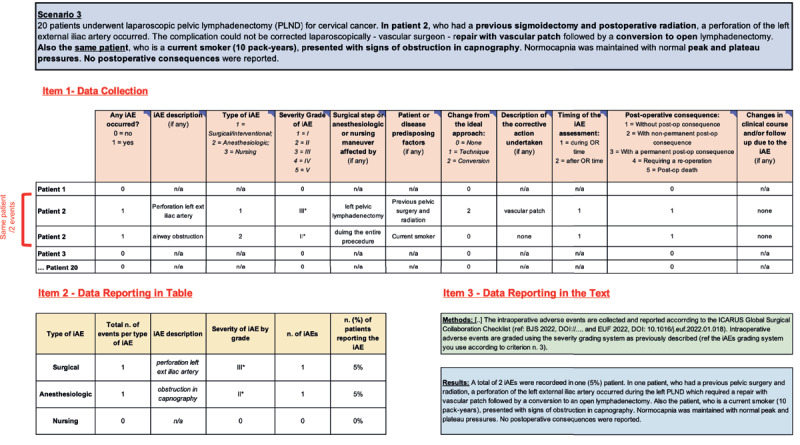
Scenario n.3: Multiple Intraoperative Adverse Events in One Patient. iAEs: Intraoperative Adverse Events; Post-op: Post-operative; n/a: Not applicable.

**Figure 5:**
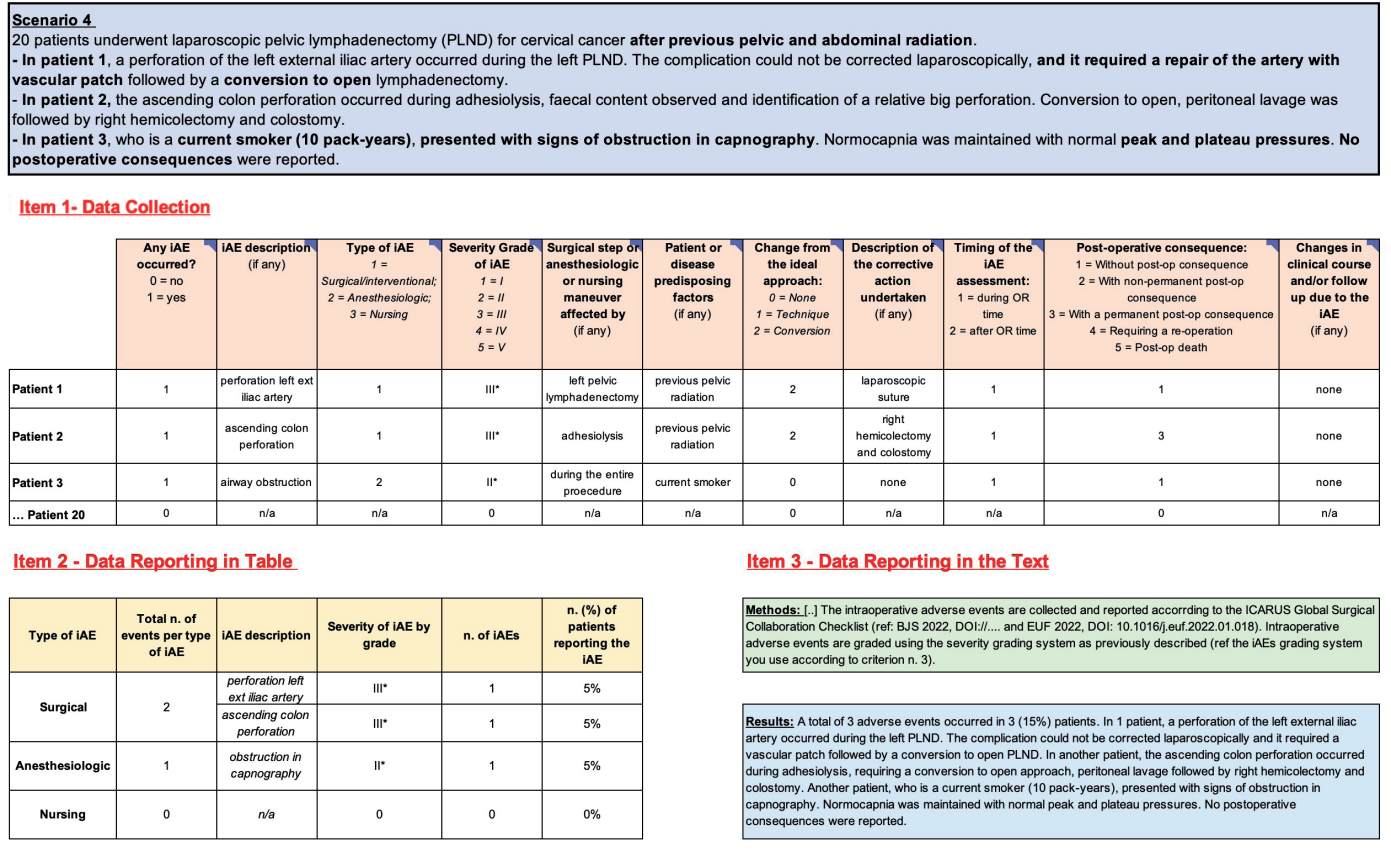
Scenario n.4: One Intraoperative Adverse Event in Different Patients. iAEs: Intraoperative Adverse Events; Post-op: Post-operative; n/a: Not applicable.

**Figure 6:**
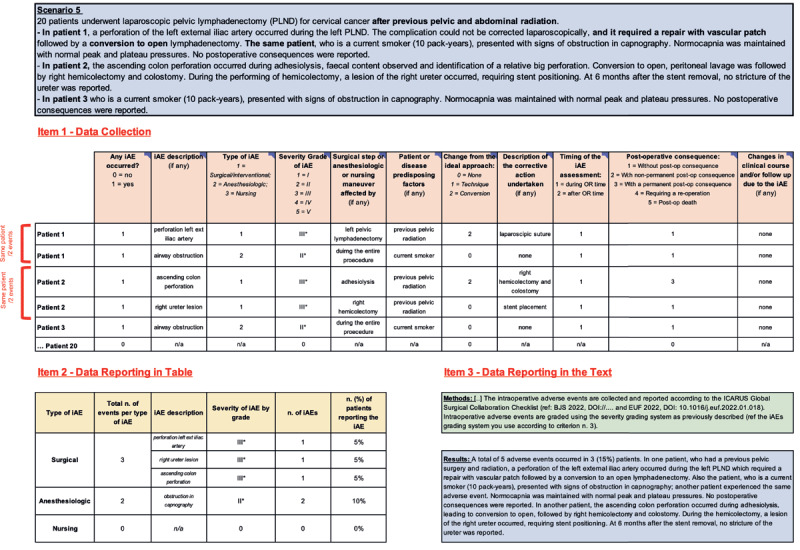
Scenario n.5: Multiple Intraoperative Adverse Events in Different Patients. iAEs: Intraoperative Adverse Events; Post-op: Post-operative; n/a: Not applicable.

### 1. Item 1 – Description of the core-set variable data collection sheet

For each iAE, a minimum core set of information should be documented to allow clinically meaningful evaluation and reporting.

This patient-by-patient core-set variable data collection sheet (**item 1**) was developed based on the 13 globally validated ICARUS criteria for reporting iAEs during and after surgical procedures [21]. These 13 criteria were distilled into the 11 core-set variables (CSVs) for properly collecting iAEs (Table 1 – Figure 1). Note that hovering your computer cursor over each core-set variable on the excel data sheet expands a description of the information to include for each core-set variable. Data variables dictionary and names are provided in supplementary materials.

**Table 1:** Core-set Variable with Corresponding Globally Validated Criteria for iAE Reporting.


CORE-SET VARIABLE DESCRIPTION	ICARUS GLOBAL SURGICAL COLLABORATION CRITERIA SATISFIED AS REFERENCE

**Any iAE occurred?** **0 = no** **1 = yes**	**Criterium 1:** In a study reporting perioperative outcomes, the intraoperative adverse events (iAEs) should be reported as one of the outcomes of interest

**iAE description (if any)**	**Criterium 2:** The intraoperative adverse events (iAEs) and the definition of each specific iAE should be reported or referenced in the methods section

**Type of iAE** ***1 = Surgical/interventional***; ***2 = Anesthesiologic***; ***3 = Nursing***	**Criterium 5:** Surgical, anesthesiology and nursing intraoperative adverse events (iAEs) should be reported separately.

**Severity Grade of iAE** ***1 = I*** ***2 = II*** ***3 = III*** ***4 = IV*** ***5 = V***	**Criterium 3:** Each intraoperative adverse event (iAE) should be reported using one of the published classification systems **Criterium 4:** Each intraoperative adverse event (iAE) should be reported separately by grade

**Surgical step or anesthesiologic or nursing maneuver affected by** **(if any)**	**Criterium 9:** The intraoperative adverse events (iAEs) should be reported specifying the surgical step or the anesthesiologic maneuver that was associated with or affected by the iAEs

**Patient or disease predisposing factors** **(if any)**	**Criterium 7:** When available, patient/disease characteristics or conditions possibly associated with intraoperative adverse events (iAEs) should be reported.

**Change from the ideal approach:** ***0 = None*** ***1 = Technique*** ***2 = Conversion***	**Criterium 8:** When the intraoperative adverse event (iAEs) requires an approach or technique conversion, both the iAEs that caused the conversion and the action undertaken should be reported.

**Description of the corrective action undertaken** **(if any)**	**Criterium 8:** When the intraoperative adverse event (iAEs) requires an approach or technique conversion, both the iAEs that caused the conversion and the action undertaken should be reported. **Criterium 11:** The management of the intraoperative adverse events (iAEs) should be reported

**Timing of the iAE assessment:** **1 = during OR time** **2 = after OR time**	**Criterium 10:** The timing of the intraoperative adverse events (iAEs) assessment should be reported as follows: **a)** If an iAE is recognized during the surgical procedure: debriefing after the surgical procedure for iAEs that are recognized intraoperatively **b)** If an iAE is not recognized during the surgical procedure report the point at which the iAE became apparent in the postoperative course

**Post-operative consequence:** **1 = Without post-op consequence** **2 = With non-permanent post-op consequence** **3 = With a permanent post-op consequence** **4 = Requiring a re-operation** **5 = Post-op death**	**Criterium 12:** The postoperative consequence of a given intraoperative adverse events (iAEs) in the postoperative course should be reported as follow: **a)** Without post-operative consequence **b)** With non-permanent post-operative consequence **c)** With a permanent post-operative consequence **d)** Requiring a re-operation **e)** Post-operative death

**Changes in clinical course and/or follow up due to the iAE (if any)**	**Criterium 13:** Report changes to the clinical course and/or follow-up that were associated with any intraoperative adverse events (iAEs)


The panel recommends including **item 1** in your database and systematically recording iAE data for every patient undergoing a surgical/interventional procedure. If a single patient experiences no iAEs, still record this data (as “zero” iAEs) to capture your population’s true iAE rate. If a patient experiences more than one iAE, the panel recommends recording the details of every iAE. To do this efficiently, simply incorporate **item 1** in your database/protocol.

The first three CSVs, (1) “any iAE occurred,” (2) “iAE description (if any),” and (3) “type of iAE (surgical, anesthesiologic, nursing)” were based on the globally validated criteria 1, 2, and 5, respectively. These variables serve to routinely name and define every AE occurring in and around the operating room, including surgical/interventional, anesthesiologic, and nursing iAEs.

CSV-4, “severity grade of iAE,” was based on ICARUS-criteria 3–4. Various methods for grading iAEs have been published [1,26,27,28,29]. The panel recommends choosing one of the severity grading systems available to maximize consistency.

CSV-5, “surgical step of or anesthesiologic or nursing maneuver affected by,” corresponds to ICARUS-criterium 9, which highlights the importance of reporting the step associated with each individual iAE. This information is needed to identify the most challenging steps associated with a specific procedure and may lead to the development of corresponding preventative/management measures.

CSV-6, “patient or disease predisposing factors,” corresponds to ICARUS-criterium 7. This information is potentially relevant for a variety of reasons. First, it could support physician decision-making; when there is a higher likelihood of an iAE given a patient risk factor, an altogether different intervention might be preferred. Second, providers do not report iAEs because of the emotional toll and fear of litigation [30]; if factors contributing to iAEs are better understood, it could create a less punitive culture.

CSVs 7–8, “change from the ideal approach” and “description of corrective action undertaken (if any),” were based on ICARUS-criteria 8 and 11. This informs whether an AE has direct patient consequences drastically changing the course of the operation. It could also ensure providers have the proper training to handle deviations from the ideal course.

CSV-9, “timing of the iAE assessment,” corresponds to ICARUS-criterium 10, and it considers when the iAE is recognized. While most iAE occurs from skin incision to skin closure, surgery is a dynamic process with opportunity for AEs before and immediately after the surgery.

CSVs 10 “post-operative consequence,” and 11 “changes in clinical course and/or follow-up due to the iAE (if any),” correspond to ICARUS-criteria 12 and 13, respectively. Both major and minor iAEs likely do not occur in isolation, potentially causing short- and long-term patient consequences. For this reason, the panel recommends completing CSVs 10–11 at patient follow-up appointments.

### 2. Item 2: Description of the summary table

This summary table is recommended to be completed once iAEs are collected and graded for each patient undergoing a specified procedure over a specified period. This will allow for clear, systematic, and transparent reporting of iAEs in single-center series and simplify any future attempts at large-scale, meta-analyses of surgical/interventional procedures as this validated tool should homogenize iAE data collection. **Item 2** distills the information collected in **item 1**, variables 1–4 (Figure 1).

Of note, ICARUS-criteria 5 calls for the separate reporting of anesthesiologic and surgical AEs. To that end, this table was designed to separately report surgical, anesthesiologic, and nursing iAEs. Furthermore, **item 2** reports the rate of AEs that occur in each of the 3 types, as well as the severity grade of each iAE.

### 3. Item 3: Description of methods and results

Following the collection of iAE information for each individual patient (**item 1**) and the completion of the summary table (**item 2**), the next step is to complete the data reporting in the text section of a research article (**item 3**). For the methods section, we have provided a succinct example that may be simply copy-and-pasted into your publication or protocol. Write the results section based on the iAEs in your group of patients. Please review the supplementary materials and the video course (https://youtu.be/pFaU7AJVLIs) for result section examples.

The results section should summarize **item 1**, distilling the information collected in the variables 1–2 and 5–11 (Figure 1). We also recommend emphasizing relevant CSVs not included in **item 2**, such as information on long-term outcomes and whether a conversion was required.

### 4. Scenarios for Completing iAE Reporting and Grading

Below are descriptions of 5 scenarios demonstrating best-practices for completing **items 1–3**. Examples have been adapted from the previously published scenario, to keep consistency [1,26,27,28,29]. An interactive video course is produced for completing **items 1–3** for the same 5 scenarios (https://youtu.be/pFaU7AJVLIs).

#### 4.1 Scenario 1: No Intraoperative Adverse Events Occurred

*Example-1: “Twenty patients underwent open right hepatectomy. None of the patients who underwent the procedure experienced any iAEs.”*

In this scenario, no iAEs occurred. In this situation, it would be reasonable to fall into the trap of thinking this information is not worthy of recording, reporting, and publishing. On the contrary, it is *essential* to systematically report and publish even if no iAEs occur, as this data provides insight into the true rate of iAEs for a given procedure.

When completing **item 1**, all columns are completed with “0” and “n/a” as there were no iAE to report/grade. The summary table (**item 2**) is completed the same way. The methods should be reported, and the results section (**item 3**) should simply state that “no iAEs occurred” (Figure 2).

#### 4.2 Scenario 2: One Intraoperative Adverse Event in One Patient

*Example-2: “Fifty patients underwent laparoscopic salvage pelvic lymphadenectomy for prostate cancer. In patient 3, who had a previous laparoscopic radical prostatectomy and postoperative radiation for high-risk prostate cancer, a perforation of the right external iliac artery occurred. The complication could not be corrected laparoscopically – vascular surgeon – repair with vascular patch followed by a conversion to open lymphadenectomy. No postoperative consequences were reported.”*

Since patients 1 and 2 experienced no iAEs, it is most appropriate to fill out the data table (**item 1**) with “0” and “n/a.” Patient 3 did experience one iAE, so you will need to input details of this event (Figure 3).

For patient 3, CSV-1 is completed with “1” as an iAE did occur. CSV-2 describes the iAE as, “perforation R ext iliac artery.” It was a surgical iAE (CSV-3), severity grade III [27](CSV-4), occurred during the right pelvic lymphadenectomy (CSV-5), required an approach conversion (CSV-7), and corrective action (vascular patch)(CSV-8). Patient history of previous pelvic surgery and radiation may have contributed to this iAE (CSV-6). It occurred during the operating room time (CSV-9) and did not result in a post-operative consequence or significant change in clinical course (CSVs 10–11). The remaining patients experienced no iAE, so complete the rest of the table with “0” and “n/a.”

Next, summarize the individual patient data in the summary table (**item 2**). Here, one patient (2%) experienced a surgical iAE. There were no anesthesiologic or nursing AE in this group of patients.

Finally, report the methods and write an original results section based on the patient outcomes. For the results section (**item 3**), be sure to include relevant information that is not included in the summary data table (**item 2**). In this scenario, examples of information to include would be patient risk factors, conversion to open, the corrective action, and the post-operative course.

#### 4.3 Scenario 3: Multiple Intraoperative Adverse Events in One Patient

*Example-3: “Twenty patients underwent laparoscopic pelvic lymphadenectomy (PLND) for cervical cancer. In patient 2, who had a previous sigmoidectomy and postoperative radiation, a perforation of the left external iliac artery occurred. The complication could not be corrected laparoscopically – vascular surgeon – repair with vascular patch followed by a conversion to open lymphadenectomy. Also, the same patient, who is a current smoker (10 pack-years), presented with signs of obstruction in capnography. Normocapnia was maintained with normal peak and plateau pressures. No postoperative consequences were reported”*.

The specific number of iAEs experienced by an individual patient should be reported. In the past, several studies reporting iAE have only highlighted whether an individual patient experienced 0 or ≥1 iAEs. This approach excludes relevant iAE information. *Scenario 3* explains how to record multiple AEs in a single patient (Figure 4).

Similarly to scenario 2, for the first iAE experienced by this patient, complete the data table as follows. CSV-1 is completed with “1” as an iAE did occur. CSV-2 describes the iAE as, “perforation left external iliac artery.” It was a surgical iAE (CSV-3), severity grade III [26](CSV-4), occurred during the left pelvic lymphadenectomy (CSV-5), required an approach conversion (CSV-7), and corrective action (vascular patch)(CSV-8). Patient history of previous pelvic surgery and radiation may have contributed to this iAE (CSV-6). It occurred during the operating room time (CSV-9) and did not result in a post-operative consequence or significant change in clinical course (CSVs 10–11).

In this example, the same patient experienced a second iAE, this one anesthesiologic in nature. When data are collected on “patient-level,” simply copy and paste **item 1** for every iAE. When data are collected on “event-level,” simply add the data regarding the second iAE on the next database raw (Figure 4). For this iAE, CSV-1 is completed with “1” as an iAE did occur. CSV-2 describes the iAE as, “airway obstruction.” It was an anesthesiologic iAE (CSV-3), severity grade II [26](CSV-4), occurred during the entire procedure (CSV-5), did not require an approach conversion (CSV-7), or corrective action (CSV-8). Patient history of current-smoker may have contributed to this iAE (CSV-6). It occurred during the operating room time (CSV-9) and did not result in a post-operative consequence or significant change in clinical course (CSVs 10–11).

Now, complete **item 2**, recording the summary data for surgical and anesthesiologic AE separately. One patient (5%) experienced a surgical iAE, and one patient (5%) experienced an anesthesiologic iAE. Finally, report the methods and write the results section (**item 3**).

#### 4.4 Scenario 4: One Intraoperative Adverse Event in Different Patients

*Example-4: “20 patients underwent laparoscopic pelvic lymphadenectomy (PLND) for cervical cancer after previous pelvic and abdominal radiation*.

*- In patient 1, perforation of the left external iliac artery occurred during the left PLND. The complication could not be corrected laparoscopically, and it required a repair of the artery with a vascular patch followed by a conversion to open lymphadenectomy*.

*- In patient 2, the ascending colon perforation occurred during adhesiolysis, faecal content was observed, and identification of a relatively big perforation. Conversion to open, peritoneal lavage followed by right hemicolectomy and colostomy*.

*- In patient 3, who was a current smoker (10 pack-years), presented with signs of obstruction in capnography. Normocapnia was maintained with normal peak and plateau pressures. No postoperative consequences were reported”*

Each iAE should be prospectively collected after each individual procedure. This should allow for greater consistency with how the data is collected across different patients receiving the same intervention. In this example, 3 patients experienced 3 unique iAEs (Figure 5).

The process for completing **item 1** is the same as already described. CSV-1 is completed with “1” as an iAE did occur. CSV-2 describes the iAE as, “perforation L external iliac artery.” It was a surgical iAE (CSV-3), severity grade III [1](CSV-4), occurred during the left pelvic lymphadenectomy (CSV-5), required an approach conversion (CSV-7), and corrective action (vascular patch)(CSV-8). Patient history of previous pelvic surgery and radiation may have contributed to this iAE (CSV-6). It occurred during the operating room time (CSV-9) and did not result in a post-operative consequence or significant change in clinical course (CSVs 10–11).

For patient 2, CSV-1 is completed with “1” as an iAE did occur. CSV-2 describes the iAE as, “ascending colon perforation.” It was a surgical iAE (CSV-3), severity grade III [1](CSV-4), occurred during the left pelvic lymphadenectomy (CSV-5), required an approach conversion (CSV-7), and corrective action (right hemicolectomy and colostomy)(CSV-8). Patient history of previous pelvic radiation may have contributed to this iAE (CSV-6). It occurred during the operating room time (CSV-9) and resulted in a permanent post-op consequence (CSV-10).

For patient 3, CSV-1 is completed with “1” as an iAE did occur. CSV-2 describes the iAE as, “airway obstruction.” It was an anesthesiologic iAE (CSV-3), severity grade II [1](CSV-4), occurred during the entire procedure (CSV-5), did not require an approach conversion (CSV-7), or corrective action(CSV-8). Patient history of current smoker may have contributed to this iAE (CSV-6). It occurred during the operating room time (CSV-9) and did not result in a post-operative consequence or significant change in clinical course (CSVs 10–11).

When completing **item 2**, note there were 2 total surgical iAEs, perforation of the left external iliac artery and ascending colon perforation. There was one anesthesiologic iAE.

Report the methods and results (**item 3**), including pertinent information based on the group of patients under study.

#### 4.5 Scenario 5: Multiple Intraoperative Adverse Events in Different Patients

*Example-5: “20 patients underwent laparoscopic pelvic lymphadenectomy (PLND) for cervical cancer after previous pelvic and abdominal radiation*.

*- In patient 1, a perforation of the left external iliac artery occurred during the left PLND. The complication could not be corrected laparoscopically, and it required a repair with vascular patch followed by a conversion to open lymphadenectomy. The same patient, who is a current smoker (10 pack-years), presented with signs of obstruction in capnography. Normocapnia was maintained with normal peak and plateau pressures. No postoperative consequences were reported*.

*- In patient 2, the ascending colon perforation occurred during adhesiolysis, faecal content observed, and identification of a significant perforation. Conversion to open, peritoneal lavage followed by right hemicolectomy and colostomy. During the performing of hemicolectomy, a lesion of the right ureter occurred, requiring stent positioning. At 6 months after the stent removal, no stricture of the ureter was reported*.

*- In patient 3 who is a current smoker (10 pack-years), presented with signs of obstruction in capnography. Normocapnia was maintained with normal peak and plateau pressures. No postoperative consequences were reported.”*

Scenario 5 is an example of reporting and grading multiple iAEs in different patients (Figure 6).

Patient 1 experienced two iAEs: a left internal iliac perforation and airway obstruction. For the perforation, CSV-1 is completed with “1” as an iAE did occur. CSV-2 describes the iAE as, “perforation L ext iliac artery.” It was a surgical iAE (CSV-3), severity grade III [1](CSV-4), occurred during the left pelvic lymphadenectomy (CSV-5), required an approach conversion (CSV-7), and corrective action (vascular patch)(CSV-8). Patient history of previous pelvic surgery and radiation may have contributed to this iAE (CSV-6). It occurred during the operating room time (CSV-9) and did not result in a post-operative consequence or significant change in clinical course (CSVs 10 and 11). For the airway obstruction CSV-1 is completed with “1” as an iAE did occur. CSV-2 describes the iAE as, “airway obstruction.” It was an anesthesiologic iAE (CSV-3), severity grade II (according to the iAE severity classification system)(CSV-4), occurred during the entire procedure (CSV-5), did not require an approach conversion (CSV-7), or corrective action (CSV-8). Patient history of current smoker may have contributed to this iAE (CSV-6). It occurred during the operating room time (CSV-9) and did not result in a post-operative consequence or significant change in clinical course (CSVs 10–11).

Patient 2 experienced two iAEs. CSV-1 is completed with “1” as an iAE did occur. CSV-2 describes the iAE as, “ascending colon perforation.” It was a surgical iAE (CSV-3), severity grade III (according to the iAE severity classification system)(CSV-4), occurred during the left pelvic lymphadenectomy (CSV-5), required an approach conversion (CSV-7), and corrective action (right hemicolectomy and colostomy)(CSV-8). Patient history of previous pelvic radiation may have contributed to this iAE (CSV-6). It occurred during operating room time (CSV-9) and resulted in a permanent post-op consequence (CSV-10). CSV-1 is completed with “1” as an iAE did occur. CSV-2 describes the iAE as, “right ureter lesion.” It was a surgical iAE (CSV-3), severity grade III [1](CSV-4), occurred during the right hemicolectomy (CSV-5), did not require a conversion (CSV-7), but did require a stent placement (CSV-8). Patient history of previous pelvic radiation may have contributed to this iAE (CSV-6). It occurred during the operating room time (CSV-9), and did not result in a post-operative consequence (CSV-10).

Patient 3 experienced one iAE. CSV-1 is completed with “1” as an iAE did occur. CSV-2 describes the iAE as, “airway obstruction.” It was an anesthesiologic iAE (CSV-3), severity grade II [1](CSV-4), occurred during the entire procedure (CSV-5), did not require an approach conversion (CSV-7), or corrective action (CSV-8). Patient history of past smoker may have contributed to this iAE (CSV-6). It occurred during the operating room time (CSV-9) and did not result in a post-operative consequence or significant change in clinical course (CSV-10 and 11). The remaining patients experienced no iAE, so complete with “0” and “n/a.”

When summarizing the data for **item 2**, note that each unique iAE is reported separately and that since the anesthesiologic iAE experienced by patients 1 and 3 were the same, only one row is needed to sufficiently report this anesthesiologic iAE. Finally, the methods and results (**item 3**) are reported based on the above information.

## Limitations

While this protocol for collecting and reporting iAE was designed to be easily accessible and widely utilized, there is potential for heterogeneity in its use and application. The specific aim of this study is to provide a composite of key variables that can be incorporated into databases all over the world with minimum effort. Variables such as demographics are usually already part of single centers or multicentric studies. The goal of producing an audiovisual component, in addition to detailed figures, was to proactively reduce alternative approaches to using this novel tool. In the future, our research team should validate the collection and reporting guidelines.

## Conclusion

We propose a core-set of variables based on globally validated criteria to standardize the collection of iAEs in surgery and anesthesiology. This tool would potentially improve interstudy reliability and consistency, and it represents the first necessary step to better understand and prevent iAEs with the goal of improving patient safety.

## Additional File

The additional file for this article can be found as follows:

10.29337/ijsp.183.s1 Supplemental Material. Core-Set of Variables for the Collection of the Intraoperative Adverse Events (iAEs) According to the ICARUS Global Surgical Collaboration Criteria.

## Abbreviations

iAEs: intraoperative adverse events

ICARUS: Intraoperative Complication Assessment and Reporting with Universal Standards CSVs: core-set variables
